# Right Ventricular Perforation From a Floating Rib Following Deep Sternal Wound Infection Debridement and Application of a Vacuum-Assisted Closure Device: A Case Report and Literature Review

**Published:** 2017-04-06

**Authors:** Amanda J. Ross, Nada N. Berry

**Affiliations:** Institute for Plastic and Reconstructive Surgery, Southern Illinois University School of Medicine, Springfield

**Keywords:** sternal wound, vacuum-assisted closure, ventricular perforation, debridement, infection

## Abstract

**Introduction:** Deep sternal wound infection following major cardiac surgery is a deleterious complication with sequelae that can be life threatening. The use of vacuum-assisted closure therapy in management of sternal wounds with resultant right ventricular rupture has been documented in the cardiothoracic and to a lesser extent in the reconstructive literature. **Methods/Case Report:** We present a case of a 67-year-old male patient who suffered from right ventricular perforation from a floating rib following debridement of a deep sternal wound infection and placement of a vacuum-assisted closure device. **Results:** Despite meticulous debridement and ensuring the release of all retrosternal adhesions, massive bleeding was encountered shortly after application of the vacuum-assisted closure device. Fortunately, quick identification of the complication and the application of direct manual pressure allowed for swift return to the operating room for repair of the defect. The patient secondarily underwent definitive closure of the mediastinal wound with an omental flap and bilateral pectoral advancement flaps. **Discussion:** Following the conclusion of this article, the reconstructive surgeon should be able to (1) identify patients at risk for ventricular perforation, (2) exhaust all means intraoperatively to prevent cardiac perforation when using vacuum-assisted closure therapy, (3) comprehend the physiology associated with vacuum-assisted closure use in this patient population, (4) have protocols in place for the management of patients with deep sternal wound infection with vacuum-assisted closure therapy postoperatively, and (5) understand basic tenets of ventricular rupture treatment should this occur to ensure prompt repair and survival.

Deep sternal wound infection (DSWI) following major cardiac surgery is a deleterious complication with sequelae that can be life threatening. As reported, the incidence of DSWIs range from 1% to 3%, with an associated mortality rate of 19% to 29%.^1^^,^^2^ The use of vacuum-assisted closure (VAC) therapy in the management of these patients has been deemed a safe and reliable option with improved survival and decreased failure rates as compared with conventional treatment.^3^^,^^4^ However, the dreaded risk of right ventricular (RV) rupture remains present with this treatment modality. The aim of this report is to highlight via literature review the prevention and management of cardiac rupture and/or perforation when using a VAC device in patients with DSWIs.

## METHODS/CASE REPORT

A 67-year-old man with a history of hypertension, hypercholesterolemia, uncontrolled diabetes, Parkinson's disease, obesity, and prior heavy tobacco use with a recent non-ST elevation myocardial infarction underwent 4-vessel coronary artery bypass grafting in a standard fashion. The patient's preoperative course was complicated by a history of angina symptoms for 6 months prior to the diagnosis of severe coronary arterial disease. In addition, the patient suffered from a non-ST elevation myocardial infarction following total knee arthroplasty 1 week prior to bypass grafting. The postoperative course was unremarkable, and the patient was discharged to a rehabilitation unit for continued physical therapy.

Approximately 3 weeks postoperatively, the patient was readmitted for purulent drainage from the sternal wound. Methicillin-sensitive *Staphylococcus aureus* was detected on wound culture, and the patient was administered antibiotic therapy. Computed tomographic scan, on admission, demonstrated an intact sternum without evidence of a fluid collection or abscess formation. The patient remained afebrile, and the white blood cell count remained within normal limits. The inferior incision was opened and packed during admission. A VAC device was placed in the wound prior to discharge.

The patient then re-presented with continued drainage, VAC failure, and acute kidney injury 2 days after discharge. Digital examination of the wound revealed continued purulent drainage and dehiscence of the inferior sternum. The patient was then urgently taken back to the operating theater for initial debridement by the cardiovascular surgery team. It was noted at the time of debridement that the sternum had dehisced completely and that the sternal wires were free floating. All sternal wires were removed in initial debridement. At this time, an intraoperative plastic surgery consultation for flap closure was requested. Upon inspection of the wound, the sternal edges were aggressively debrided on the left hemisternum, including a portion of the rib that became free floating from the sternal edge. Inspection of the mediastinum displayed no adherence to the anterior RV. The soft tissues were pale and wound defect was large in the barrel-chested patient. The decision was made to continue debridement and to temporize the wound with a VAC device before final closure, as the wound required additional tissue transfer for dead space closure. Polyurethane foam material was placed in the defect in a 2-layer fashion. A Mepitel (Molnlycke Health Care, Dunstable, United Kingdom) underlay was placed on the posterior aspect of the polyurethane foam to protect the vital structures from direct contact with the sponge. The VAC was set to negative 50 mm Hg and found to have adequate seal prior to termination of the procedure.

Nine hours postoperatively, the patient was moved from his hospital bed to his chair with his shoulders in an adducted position. Following these maneuvers, the patient experienced an episode of bleeding into the VAC canister of approximately 700 mL of frank blood. A blood transfusion was initiated immediately. Bedside suction and dressing supplies were made available. The VAC was removed at the bedside by the plastic surgery team. After gentle removal of large intrathoracic hematoma, bright red blood under high pressure coming from the anterior heart surface was identified. Manual pressure was held over the heart Ventricular perforation was suspected, and the patient was emergently taken to the operating room (OR) and placed on cardiopulmonary bypass by the cardiothoracic surgeon.

## RESULTS

The diagnosis of RV perforation from the floating rib was confirmed, and the defect was repaired using pledgets. The wound was tightly packed with saline gauze dressings. Unfortunately, in the postanesthesia care unit (PACU), the patient again experienced bleeding, requiring a reinforcement of the ventricular repair with additional pledgets in the OR. After reinforcement, the sternal defect was definitively closed with an extra-abdominal pedicled omental flap and bilateral myocutaneous pectoralis major advancement flaps. The patient was discharged to a rehabilitation unit 2 weeks postoperatively without major complication after flap closure.

## DISCUSSION

The use of VAC therapy in management of DSWI and RV rupture has been well documented in the cardiothoracic^3^^-^7 and to a lesser extent in the reconstructive literature. Education on the prevention and management of cardiac perforation is paramount to the reconstructive surgeon. We aim to use our case presentation to highlight learning points that should be discussed within each reconstructive team prior to the treatment of these patients. It is imperative that the reconstructive surgeon be able to (1) identify patients at risk for ventricular perforation, (2) exhaust all means intraoperatively to prevent perforation when using VAC therapy in DSWI patients, (3) comprehend the physiology associated with VAC use in this patient population, (4) have protocols in place for the management of DSWI patients with VAC therapy postoperatively, and (5) understand basic tenets of ventricular rupture treatment should this occur to ensure prompt repair and survival.

### Identifying patients at risk for cardiac rupture

For the reconstructive surgeon, it is important to know the full history of the patient and the time line of important medical and surgical events prior to debridement. Events such as recent myocardial infarction affecting the free wall of the right ventricle, bypass surgery, types of bypass grafts used, foreign material in the operative field, the onset of infection symptoms, and chronic RV dilatation are important to consider when assessing myocardial health. Cartier at al^8^ recognized that of 48 patients with postoperative mediastinitis, 7 of whom went on to develop RV rupture, obesity with a body mass index greater than 30 kg/m^2^ and infection identification delay were risk factors that were statistically linked to the occurrence of RV rupture. In addition, Arbulu et al^9^ noted that weakness in myocardial wall from previous insult, such as an infarction, may cause avulsion trauma to the RV to occur more readily. Edwin^9^ emphasized that mechanical sternal trauma from an open or dehisced sternum, akin to a rip saw effect on the adjacent RV wall, can promote spontaneous rupture. Even armed with this information, Edwin^10^ goes on to warn that a very thin RV wall may rupture without any mechanical sternal contributions whatsoever. Assessing a patient's preoperative state will assist in operative planning and counseling of the patient on his or her unique risk factors that could contribute to rupture.

### Intraoperative debridement and VAC application

In regard to intraoperative prevention of RV rupture, tenets of sternal wound debridement are straightforward. Arubul et al^9^ stress 3 key steps in initial operative management: (1) limit open packing and close with flaps as soon as possible based on clinical evaluation; (2) free adhesions between the anterior RV and sternal halves to prevent avulsion; and (3) sternal edges should be meticulously smoothed after debridement. In addition, the use of antimicrobial irrigation, such as betadine, Dakin's, and other antibiotics, intraoperatively may increase desiccation risk and further weaken the myocardial wall.^9^ While these steps were formulated for the “open” method of treatment, nevertheless their significance can be extrapolated to those patient treated with a negative pressure wound therapy. In a study by Gustafsson et al,^11^ 40 patients with DSWI were treated with sternal-sparing debridement technique in conjunction with VAC therapy. All patients received sternal reconstruction without the use of muscle or omental flap surgery, highlighting a conservative and efficacious approach to sternal wound debridement.^11^

When using conventional VAC therapy to temporize a sternal wound, placement of the polyurethane foam and vacuum setting must be taken into consideration. Two goals are kept in mind when applying the VAC. First, one must successfully cover the heart. Second, the sternum must be fixated. Protective covering, such as 3 to 4 layers of paraffin gauze sheets, such as Adaptic (Acelity, San Antonio, Tex), can be used to line the lungs, heart, and bypass grafts to prevent direct contact with the polyurethane foam. This reduces negative pressure from acting directly on the heart and prevents adherence between the sternum and the RV. The foam should then be placed in a 2-layer fashion, with the posterior piece at the level of the sternum and the anterior piece at the level of the skin.^5^^,^^11^ It is the authors’ recommendation that VAC sponge selection can utilize the less adherent white foam for the posteriorly placed piece whereas the anterior piece can utilize either the standard black sponge or antimicrobial silver sponge. While no studies have confirmed the exact optimal negative pressure for VAC treatment of sternotomy wounds, Malmsjö et al^12^ endorse the use of negative 75 to 125 mm Hg. These settings are used to mimic physiologic conditions of a normal chest cavity and to provide the most effective stability to the sternum. It is postulated that the use of intermittent negative pressure therapy is unsuitable for the treatment of sternal wounds.^12^

Recently, a series of articles from Malmsjö et al^12^ has investigated the specific physiologic nature of RV rupture during VAC treatment. It is believed that rupture may be potentiated by repeated trauma from contact of the RV with sharp sternal edges. In their porcine model, as little as 50 mm Hg of pressure contact was sufficient to cause damage to the anterior surface of the heart.^13^ In light of this, Ingmansson and colleagues^14^ have developed a novel device used to protect the heart from the sternal edges, aptly named the HeartShield. This device consists of a T-shaped plastic form covered with foam and low-adherence wound contact layer. This system can be used in combination with conventional VAC therapy. Results from a small cohort of patient studies are promising.^14^

### Protocols for patients with DSWI treated with VAC therapy

While, traditionally, the closure of sternal wounds after debridement has been based on clinical evaluation of granulation tissue, other parameters may be considered in regard to correct timing of the closure. The Lund University Hospital mediastinitis algorithm published by Sjögren et al^15^ reports successful delayed primary closure without the use of free tissue transfer in VAC patients. The algorithm advocates initial sternal-sparing debridement, gentle saline rinse, and application of continuous-suction VAC at negative 125 mm Hg. Vancomycin and imipenem are used for initial antibiotic treatment. After 2 to 4 days, C-reactive protein (CRP) levels are monitored. If the value is less than 70 mg/L, delayed primary closure, including sternal rewiring, is attempted. If the defect is large, soft-tissue flap surgery is employed. Antibiotics are continued for 6 weeks. If CRP values are greater than 70 mg/L, the VAC is removed in the OR, and the wound is recultured to guide antibiotic therapy and debrided. VAC therapy continues for another 2- to 4-day cycle, and CPR levels are reexamined to determine whether closure can occur.^15^ A comparison of 30-day mortality data demonstrates that the benefits of microbiological control with repeated debridement outweighed the risk of RV rupture in 176 patients treated for DSWI with VAC therapy.^5^

Postoperatively, abrupt changes in intrathoracic pressure should be avoided so as to decrease the risk of sheering forces. Efforts should be made to quell vigorous coughing and episodes of postoperative nausea and vomiting. The use of a postoperative soft corset/chest harness is strongly recommended, especially in obese patient or those at risk for postoperative coughing such as patients with chronic obstructive pulmonary disease.^5^^,^^15^ If the patient remains intubated, close monitoring for signs that the patient is “fighting the ventilator” should be undertaken.^9^

While a properly placed VAC does facilitate early mobilization, communication between the surgeon and the nursing/physical therapy staff must be clear to delineate restrictions in motion. To date, there have been no reported accepted clinical guidelines for timing of mobilization in patients with unstable sternums following DSWI debridement. However, recently, Cahalin et al^16^ addressed revisions to historical sternal precautions in order to maximize benefits for optimal recovery while decreasing functional impairment in patients with a median sternotomy. For high-risk patients, such as those with unstable sternums, a sternal precaution algorithm was developed. For 2 to 4 weeks after surgery, the algorithm proposes that patients: (1) participate in no lifting, pushing, or pulling more than 10 lb; no shoulder abduction or flexion greater than 90° when the upper extremities are weighted; (2) participate in shoulder active range of motion in a “pain-free” range; (3) have no scapular retraction past neutral; (4) have limited active trunk flexion/rotation with movements from the supine to sitting positions; (5) have no use of upper extremities with sitting to standing; and (6) continue the use of a sternal splint/compression device to mitigate effects of coughing or Valsalva maneuvers.^16^ We caution that aggressive shoulder adduction and/or abduction should be avoided in the early postoperative period. Ideally, a patient should be monitored overnight with bed rest precautions and carefully evaluated by physical therapy in the first few days postoperatively for mobilization readiness.

### Tenets of management after ventricular rupture

Notification should be immediately sent to the cardiovascular OR team and the cardiothoracic surgeon if a rupture is suspected. We advocate that all patients be typed and screened for blood products before undertaking any deep sternal wound debridement. Should rupture occur, the administration of intravenous fluids and blood products for immediate resuscitation should be initiated as soon as possible. In addition, the VAC appliance should be shut off to relieve further negative pressure on the myocardium and the appliance should be removed at the bedside with a senior surgeon present. Prior to VAC removal, functional wall or bedside suction should be set up and packing material such as sponges and gauze roll be readily available. Exploration of the wound and visualization of the source of bleeding can then be safely undertaken. Application of manual pressure to the tear site should be performed once identified. Once an OR is secured, the patient may require cardiopulmonary bypass for large ruptures.

As noted in the cardiac literature, there are multiple modalities for repair including suture repair with Dacron pledgets, pericardial patches, fascia lata, TachoComb sheets (equine collagen patch with human fibrinogen and human thrombin; CSL Behring, Tokyo, Japan), and Teflon felt patch with suture anchor or cyanoacrylate glue.^8^^,^^17^ Discussion between the cardiovascular and reconstructive teams should include methods and feasibility of repair based on the appearance of native tissues. Consideration of flap closure at this time should be weighed heavily for further protection of the repair. Monitoring in the intensive care unit postoperatively is advocated for potential rebleeding.

## CONCLUSION

Using VAC therapy in the setting of DSWI is appropriate and beneficial as compared with conventional treatment methods. The risk of RV rupture still exists in this population, and its cause can be multifactorial. As in the case of our patient, presurgical cardiac ischemia, a history of obesity, prolonged infection, and discovery of dehiscence of the sternum with free floating sternal wires within the wound could potentially increase the risk of damage to the myocardium prior to any surgical washout. Intraoperatively, adhesions were freed and the sternal edges were meticulously debrided prior to VAC placement. While the underlay material differed, we hypothesize that this did not have a large impact on impending perforation. Rather, a mix of aggressive debridement of the sternum with resulting free floating rib, instability of the remaining bony components, as well as an increase in intrathoracic pressure not controlled by the low negative pressure setting of the VAC likely led to rupture. Postoperative protocols to decrease intrathoracic pressure were not in place and further added to impending perforation. Notification of massive bleeding into the VAC prompted the quick response of the reconstructive attending surgeon. Appropriate blood products were ordered, the VAC was removed at the bedside, and manual pressure was held until the cardiothoracic team arrival and the OR preparation. Communication between the 2 surgical teams was paramount. Fortunately, for our patient, despite 2 episodes of bleeding, omentum and bilateral pectoralis advancement flaps filled the dead space successfully and no further complications were encountered.
